# Resting-State Cerebello-Cortical Dysfunction in Parkinson's Disease

**DOI:** 10.3389/fneur.2020.594213

**Published:** 2021-01-28

**Authors:** William C. Palmer, Brenna A. Cholerton, Cyrus P. Zabetian, Thomas J. Montine, Thomas J. Grabowski, Swati Rane

**Affiliations:** ^1^Department of Radiology, University of Washington Medical Center, Seattle, WA, United States; ^2^Department of Pathology, Stanford University School of Medicine, Stanford, CA, United States; ^3^Geriatric Research Education and Clinical Center, Veterans Affairs Puget Sound Health Care System, Seattle, WA, United States; ^4^Department of Neurology, University of Washington School of Medicine, Seattle, WA, United States

**Keywords:** Parkinson's disease, cerebellum, BOLD fMRI, resting-state connectivity, resting-state networks

## Abstract

**Purpose:** Recently, the cerebellum's role in Parkinson's disease (PD) has been highlighted. Therefore, this study sought to test the hypothesis that functional connectivity (FC) between cerebellar and cortical nodes of the resting-state networks differentiates PD patients from controls by scanning participants at rest using functional magnetic resonance imaging (fMRI) and investigating connectivity of the cerebellar nodes of the resting-state networks.

**Materials and Methods:** Sixty-two PD participants off medication for at least 12 h and 33 normal controls (NCs) were scanned at rest using blood oxygenation level-dependent fMRI scans. Motor and cognitive functions were assessed with the Movement Disorder Society's Revision of the Unified Parkinson's Disease Rating Scale III and Montreal Cognitive Assessment, respectively. Connectivity was investigated with cerebellar seeds defined by Buckner's 7-network atlas.

**Results:** PD participants had significant differences in FC when compared to NC participants. Most notably, PD patients had higher FC between cerebellar nodes of the somatomotor network (SMN) and the corresponding cortical nodes. Cognitive functioning was differentially associated with connectivity of the cerebellar SMN and dorsal attention network. Further, cerebellar connectivity of frontoparietal and default mode networks correlated with the severity of motor function.

**Conclusion:** Our study demonstrates altered cerebello-cortical FC in PD, as well as an association of this FC with PD-related motor and cognitive disruptions, thus providing additional evidence for the cerebellum's role in PD.

## Introduction

Parkinson's disease (PD), a common progressive neurodegenerative disorder, is characterized primarily by motor symptoms but also has cognitive symptoms. PD traditionally has three pathological hallmarks (1). The first is the progressive loss of dopaminergic neurons in the substantia nigra. Second, the result of nigral neuronal death is a marked depletion of dopamine in the striatum, which has been the principal target for treatment. Third, PD is indicated by the presence of Lewy bodies, composed of α-synuclein aggregates, in the nigra and other subcortical and cortical regions. Neuroimaging studies have played a critical role in our understanding of how these trademark pathologies influence neuronal function in humans (2). However, most studies have focused on the cortex, even though the cerebellum has also been implicated in the disease state of PD (3).

Studies that have investigated the cerebellum demonstrate that PD patients have cerebellar atrophy (4, 5) and hyperactivity (6, 7). Resting-state functional connectivity (FC) studies have also uncovered abnormalities in the cerebellum. Initial studies have examined the cerebellum's primary output, the dentate nucleus, or motor regions of the cerebellum, such as lobules V and VI (8, 9). These studies suggest atypical connectivity in PD patients within the various cerebellar regions, as well as between the cerebellum and the cortex (prefrontal, parietal, and temporal) and subcortical areas of the motor system. However, the cerebellum has been demonstrated to be involved in tasks beyond motor functioning, such as cognitive and affective processes, and non-motor symptoms are observed in PD patients (1, 10). Cerebellar involvement in cognitive functioning is supported by supramodal zones, particularly crus I and II, that are functionally connected to association areas, such as prefrontal and posterior-parietal cortex (11, 12).

Recent studies have explored the FC of larger portions of the cerebellum. In a network investigation utilizing whole cerebellar parcellations defined by anatomical boundaries, PD patients showed increased positive connectivity between somatomotor regions of the cerebellum and somatomotor cortical areas compared to controls (13). Furthermore, abnormal subcortical connectivity was discovered within the cerebellum and between the cerebellum and reward system (nucleus accumbens and orbitofrontal regions). Weaker correlations between the striatum and somatomotor cerebellar regions have also been discovered previously in PD patients compared to controls (14). Impaired visuospatial performance was associated with decreased positive intracerebellar connectivity, decreased magnitude of negative cerebellar to visual network FC, and a switch from negative cerebellar to reward FC in controls to positive in PD (13). Other studies investigated cerebellar regions that combine multiple anatomical territories. Analyses using clustering methods have uncovered unusual FC within identified cerebellar networks and between these networks and cortical areas including occipital, parietal, and frontal cortices in PD patients (15, 16). In a seed-based analysis, O'Callaghan et al. (17) explored a sensorimotor subregion that included lobules V, VI, VII, and VIII and a cognitive subregion that consisted of Crus I and II. This study probed the cerebellum's FC to large-scale cortical resting-state networks (RSNs) and cerebellar atrophy. PD patients had decreased FC between the cognitive cerebellum and somatomotor network (SMN). Cerebellar atrophy was correlated with changes in FC between the cerebellum and SMN, default mode network (DMN), dorsal attention (DAN), and frontoparietal network (FPN). Finally, the Movement Disorder Society's Revision of the Unified Parkinson's Disease Rating Scale III (MDS-UPDRS III) score was negatively correlated with cerebellar atrophy and FC between both motor and cognitive cerebellum and the SMN. These correlations between cerebellar FC and PD test scores are evidence that the cerebellum is implicated in the neurophysiology of PD and may thus have potential as a therapeutic target.

We sought to perform a network-based seed analysis of the cerebellum's FC to the cerebral cortex. Parcellations of the cerebellum were determined by Buckner's 7-network cerebellar atlas, which defines cerebellar nodes of the dominant cerebral RSNs (12). These seeds were not segregated by lobule, but rather by their proper cortical RSNs. Lobular boundaries have been found to be inconsistent with functional subdivisions in the cerebellum (18). Instead of seeding the cortical RSNs, we used the Buckner cerebellar atlas to seed the cerebellum for FC. To validate the cerebellar seeds, we confirmed the RSNs generated by these seeds. We hypothesized that such analysis would continue to show differential FC between PD patients and controls especially in motor areas of cortex. We also investigated whether performance on non-motor tests was correlated with cerebello-cortical FC.

## Materials and Methods

### Participants

This study included 95 participants, who were recruited from the Pacific Udall Center between 2016 and 2019. All participants underwent a full neuropsychological battery [including the Montreal Cognitive Assessment (MoCA)], a neurological examination, and the MDS-UPDRS III. Data from these assessments were reviewed at a diagnostic consensus conference, attended by at least two movement disorders neurologists and a neuropsychologist, to determine the diagnostic category [PD or normal control (NC)] and cognitive status [normal, mild cognitive impairment (MCI), dementia]. PD with dementia required classification of parkinsonism prior to dementia by at least 1 year to exclude patients with dementia with Lewy bodies. Other exclusion criteria included individuals who had pathogenic mutations other than apolipoprotein E (APOE) and glucocerebrosidase (GBA) [e.g., leucine-rich repeat kinase 2 (LRRK2), α-synuclein (SNCA), Parkinson's Disease gene (PARK)2, phosphatase and tensin homolog-induce dkinase 1 (PINK1), and PARK7] and individuals who had contraindications to MRI (e.g., pacemaker or claustrophobia) or were non-ambulatory. Complete descriptions of this process and diagnostic criteria have been detailed previously (19–21). Sixty-two participants were determined to meet UK Parkinson's Disease Society Brain Bank clinical diagnostic criteria for PD, and 33 participants were classified as NC without PD symptoms. Prior to the scan, PD patients had not taken their dopamine replacement therapy for at least 12 h. During the scan, participants were directed to keep their eyes open and fixate on a cross without thinking of anything in particular. MDS-UPDRS III was administered on the same day as the scan without dopamine replacement therapy for PD patients. Within 6 months of imaging, MoCA scores were also collected for each participant. MDS-UPDRS III and MoCA scores were used as measures of motor dysfunction and cognitive performance, respectively (22, 23). Higher scores on MDS-UPDRS III indicate more severe motor symptoms, while higher scores on the MoCA imply better cognitive performance.

### Imaging

All scans were acquired on a Philips 3T Achieva scanner with a 32-channel head coil using Sensitivity Encoding Reception. Anatomical T1 weighted scans were 3D MPRAGE sagittal acquisition. Spatial resolution was 1 × 1 × 1 mm^3^, repetition time (TR)/echo time (TE) was 10.17/4.71 ms, and inversion time (TI) was 900 ms. The blood oxygen level-dependent (BOLD) functional MRI (fMRI) acquisition was a whole-brain 2D-echo planar imaging acquisition. Resolution was 3.5 × 3.5 × 3.5 mm^3^, matrix size was 64 × 64 mm^2^, and TR/TE was 2,500/45.5 ms. Each fMRI run acquired 240 time points of data.

### Analysis

Preprocessing was completed using FSL (v5.0), AFNI (v17.3), and SPM12 (24–26).

#### Anatomical Image Processing

The anatomical T1 images were segmented into gray matter (GM), white matter (WM), and cerebrospinal fluid (CSF) after brain extraction. Average GM probability was calculated from the segmented GM probability partial volume estimate.

#### Seed-Based fMRI Connectivity Analysis

First, fMRI data were motion corrected. Participants with motion >1 fMRI voxel were excluded from the study. Next, baseline drift was removed with a 0.01-Hz high pass filter in FSL. To remove extreme motion, despiking was conducted in AFNI. The remaining preprocessing and analysis were conducted in Conn (v18). Functional data were co-registered to corresponding T1-weighted anatomical images in 2-mm Montreal Neurological Institute (MNI) space and normalized to the standard space. The anatomical CompCor algorithm was used to remove physiological noise using masks of WM and CSF (27). Briefly, the masks are thresholded to ensure that mask voxels contain at least 99% WM or 99% CSF. The CompCor routine in Conn then extracts the time courses from the WM and CSF voxels in the mask, constructs the covariance matrix, and performs principal component analysis (PCA). The most significant components from the PCA are extracted and used as estimates of physiological noise. These components were then used as confounds and regressed from the BOLD time series. Lastly, smoothing was performed with an 8-mm full-width half-maximum kernel. Based on our voxel size, a kernel of 8 mm is appropriate to reduce bias, ensure homoscedasticity, and improve signal-to-noise ratio for group-level analysis (28, 29). We do not expect the voxel or smoothing kernel size to negatively impact our results, as our seeds (~24–75 cc) are much larger than both the voxel size (~0.043 cc) and smoothing kernel (0.512 cc). Quality assurance involved manual inspection of data after each preprocessing step. For a flowchart of software and commands used for each step, see Supplementary Figure 1.

**Figure 1:**
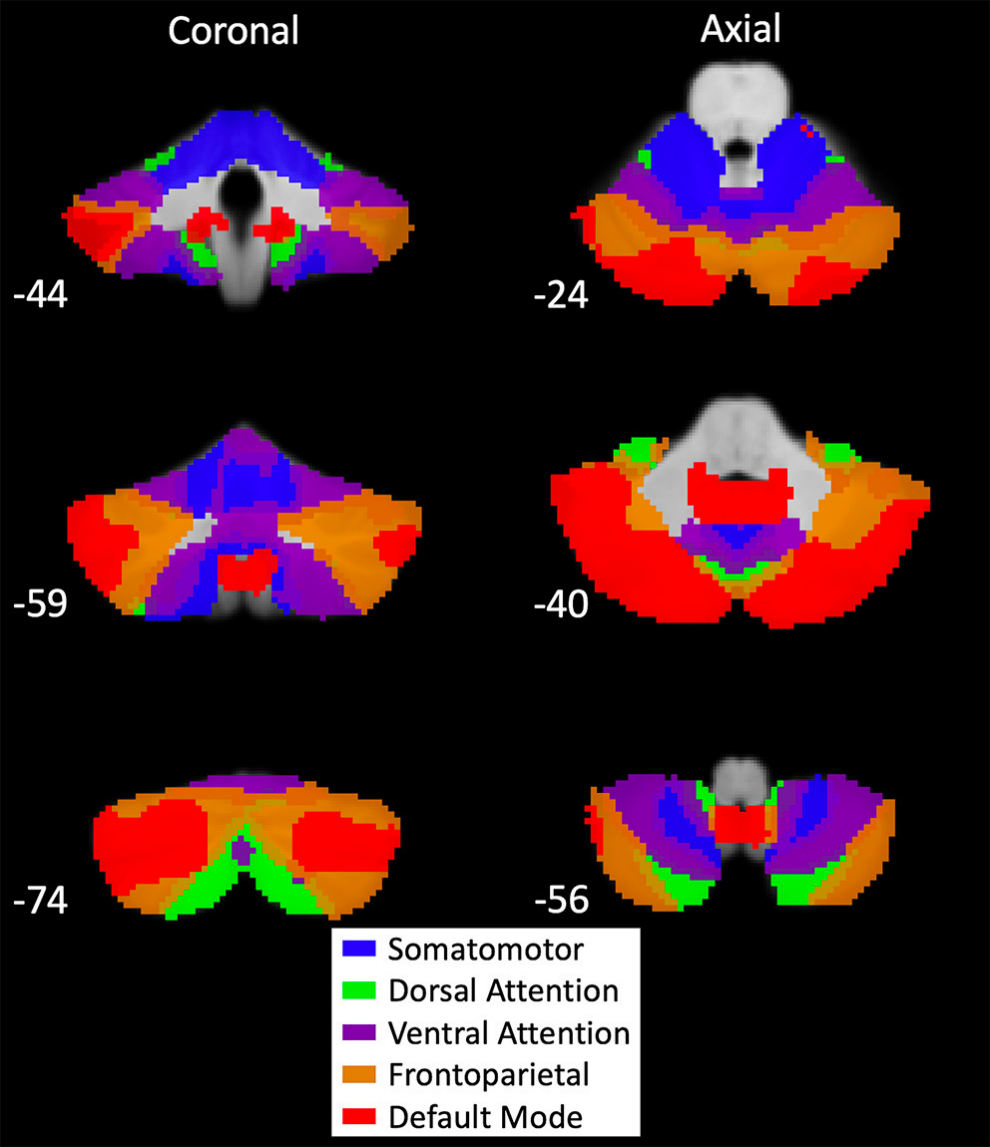
Buckner's 7-network cerebellar atlas seeds. Map of cerebellar seeds produced by functional connectivity of large-scale resting-state networks to the cerebellum (12). Montreal Neurological Institute (MNI) coordinates are provided in the bottom left of each section. Networks included in our analysis were the somatomotor (blue), dorsal attention (green), ventral attention (purple), frontoparietal (orange), and default mode (red). Visual and limbic networks were excluded from analysis because only motor and cognitive functions were examined. These network seeds have multiple distinct regions of interest (ROIs) on the cerebellum. Average time series for seed-based analysis were generated using all ROIs within a network.

A seed-to-voxel whole-brain analysis was performed using the cerebellar parcellations defined by Buckner's 7-network atlas (12). The networks included the SMN, FPN, DMN, DAN, ventral attention, visual, and limbic networks. For this study, the visual and limbic networks were excluded because our interests were in investigating motor and cognitive functioning. Additionally, the SMN, FPN, DMN, and DAN have mainly been implicated in PD (30). The cerebellar networks are larger than the traditional spherical regions of interest (ROIs). Our seeds are network seeds with multiple distinct ROIs on the cerebellum, such as bilateral ROIs (Figure 1). The averaged time series of all the ROIs within the network seeds was obtained for seed-to-voxel connectivity analysis. Connectivity was measured as the correlation between the average time series of all voxels within a cerebellar network ROI and the time series of each voxel in the brain. While Buckner's atlas only considered positive correlations, both positive and negative correlations were used in this study. Statistical tests consisted of differences between groups, main effects of MDS-UPDRS III and MoCA scores, and interaction between MoCA score and group condition. Generalized linear models were adjusted for age, gender, average motion, and average GM probability. For all comparisons, we applied a two-sided voxel-wise thresholding at *p* = 0.005 and a cluster-size family-wise error (FWE) correction for multiple comparisons at *p* = 0.05.

Buckner's cerebellar atlas was derived from cortical RSNs. In this study, we seeded the cerebellar nodes of the RSNs to identify connectivity in cortical regions. Therefore, we first investigated the average connectivity of each cerebellar node to demonstrate it would reproduce the corresponding RSN (Supplementary Figure 2). Additionally, some previous studies chose lateralized ROIs in the cerebellum, and evidence of distinct functions of the left and right cerebellum exists (10). Hence, we split the SMN cerebellar seed by hemispheres and seeded both to investigate if our results are robust across hemispheres or have lateralized differences.

## Results

### Clinical and Demographic Data

Fifty-seven PD participants (male = 40; female = 17) and 30 NC participants (male = 19; female = 11) were included in the final analysis. During quality assurance steps, eight participants were removed due to excessive motion or poor registration. Final analysis included six PD patients with dementia and 23 with MCI. On the other hand, no NC participants had dementia, but nine had MCI. While PD participants were 67.2 ± 8.1 (mean ± standard deviation) years old, NC participants were 70.7 ± 8.8 years old; the difference was not significant (*p* = 0.07). Among PD patients, the average MDS-UPDRS III score was 31.1 ± 12.1. Both PD (26.5 ± 2.6) and NC (26.6 ± 2.6) participants scored similarly on the MoCA, and the difference was not significant (*p* = 0.87). However, the difference in GM probability between PD (69.6 ± 1.3%) and NC (70.1 ± 1.3%) participants was significant (*p* = 0.05). This was expected because atrophy is well documented in PD (4, 5). In order to ensure that the FC differences exist despite the GM atrophy, the GM probabilities were included as a nuisance regressor in all subsequent analyses.

To assess for potential differences between PD and NC participants in movement during the scans, we compared motion between groups. Since participants with excess motion (>voxel size) were eliminated, the average motion (along X, Y, and Z axes) was 0.23 ± 0.34 mm in PD patients and 0.17 ± 0.13 mm in NC participants. This difference between groups was not statistically significant (*p* = 0.26). Still, the average motion estimates were included as a nuisance regressor to minimize the association of FC with motion. A summary of all demographic and clinical data is located in Table 1. For clarity of all FC analyses, cortical RSNs are denoted with abbreviations as above (e.g., SMN), while cerebellar components are denoted with a prefix “c” (e.g., cSMN).

**Table 1:** Table of demographic and clinical data.

**Condition**	**Male**	**Female**	**Age**	**Dementia**	**MCI**	**No CI**	**MDS-UPDRS III**	**MoCA**	**Motion (mm)**	GM Prob* (%)
PD	40	17	67.2 ± 8.1	6	23	28	31.1 ± 12.1	26.5 ± 2.6	0.23 ± 0.34	69.6 ± 1.3
NC	19	11	70.7 ± 8.8	0	9	21	—	26.6 ± 2.6	0.17 ± 0.13	70.1 ± 1.3

Data are stratified by condition (Parkinson's disease participants, PD; normal control, NC). Clinical data include cognitive diagnosis, which has three levels: dementia, mild cognitive impairment (MCI), and no cognitive impairment (No CI). The last two columns display average motion and gray matter probability in the two groups. Individual values were used as nuisance regressors in our analysis. All means are given with standard deviation (mean ± standard deviation). Other values are counts of participants belonging to that group. An asterisk indicates statistically significant differences (*p = 0.05). MoCA, Montreal Cognitive Assessment; MDS-UPDRS III, Movement Disorder Society's Revision of the Unified Parkinson's Disease Rating Scale III.

### Group Differences Between Parkinson's Disease Patients and Controls

**Figure 2:**
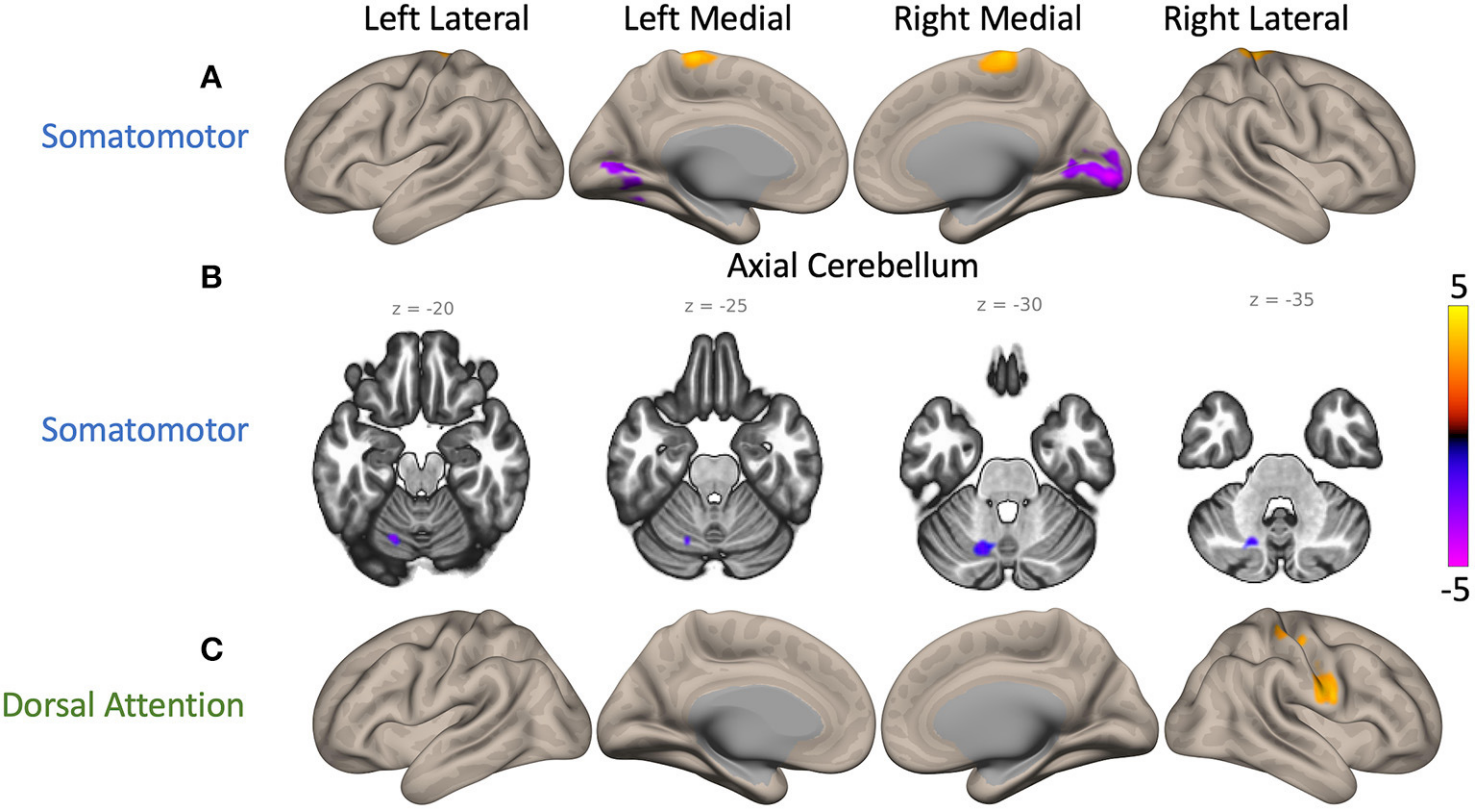
Maps of cerebellar functional connectivity (FC) differences between Parkinson's disease (PD) patients and normal controls (NCs). Significant differences were found when seeding the cerebellar nodes of the somatomotor network (cSMN) and dorsal attention network (cDAN). The color bar represents z-scores, with yellow displaying where PD patient FC values were greater than NC participant values. **(A)** Two cerebral clusters were observed when seeding the cSMN. Compared to NC participants, PD patients showed greater FC between the cSMN and the precentral gyrus (*p* = 0.01, yellow) and lower FC between the cSMN and the right intracalcarine cortex (*p* = 0.0004, purple). **(B)** Another cluster located within lobule VI, crus I, and lobule VIII on the left side had lower FC with the cSMN in PD participants compared to controls (*p* = 0.0004, purple). **(C)** PD participants also had greater FC between the cDAN and the right postcentral gyrus than controls (*p* = 0.004, yellow). An average cortical connectivity map for all subjects can be found in Supplementary Figure 2.

Group differences were identified in FC of cSMN and cDAN to cortex. Compared to NC participants, PD patients had higher FC between the cSMN and the bilateral precentral gyrus, postcentral gyrus, and SMA (*p* = 0.01, depicted by yellow cluster in Figure 2A). PD patients had lower FC between the cSMN and the occipital lobe, specifically, bilateral intracalcarine cortex, occipital pole, lingual gyrus, right supracalcarine cortex, left occipital fusiform cortex, and temporal occipital fusiform cortex (*p* = 0.0004, purple cluster in Figure 2A), compared to controls. Furthermore, PD patients had lower FC between the cSMN and the lobule VI, crus I, and lobule VIII on only the left side (*p* = 0.0004, purple cluster in Figure 2B). PD participants also had higher right lateralized FC between the cDAN and the right post-central gyrus, pre-central gyrus, and superior parietal lobule (*p* = 0.004, yellow cluster in Figure 2C) compared to NC participants.

Similar differences were found when seeding the left and right hemispheres of the cSMN. For the left cSMN, there was higher FC to bilateral pre-central gyrus, SMA, and right post-central gyrus (*p* = 0.01, yellow cluster in Supplementary Figure 3B) but lower FC to occipital lobe (*p* = 0.005, purple cluster in Supplementary Figure 3B). On the other hand, FC for the right cSMN did not survive FWE correction at 0.05 but was evident at 0.07. With FWE correction at 0.07, right cSMN also had higher FC to bilateral precentral gyrus, postcentral gyrus, and SMA but lower FC to occipital lobe (Supplementary Figure 3C). These results support that our findings for the cSMN are robust and there are no major lateralized differences between the FC of the left and right cSMN.

In summary, compared to NC participants, PD patients had higher FC between the cSMN and the frontal regions of the cortex but lower FC from the cSMN to the posterior regions of the cortex. Furthermore, they had higher FC between the cDAN and the cortical somatomotor areas.

### Main Effects of Movement Disorder Society's Revision of the Unified Parkinson's Disease Rating Scale III and Montreal Cognitive Assessment Scores

**Figure 3:**
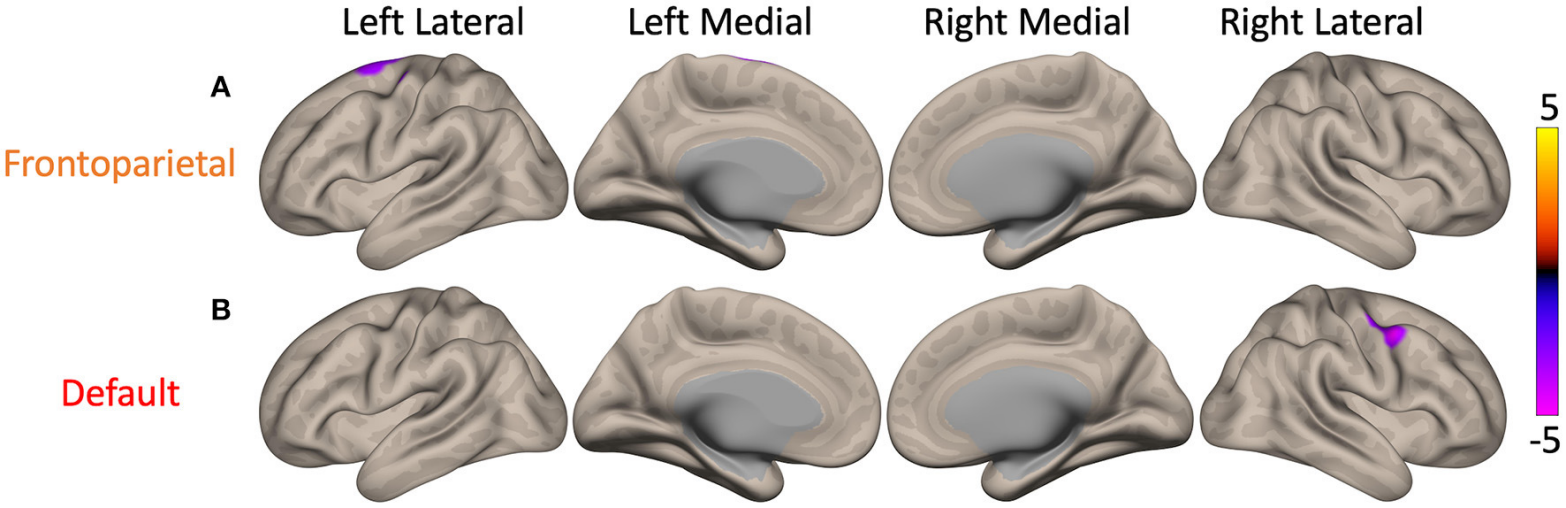
Maps of the main effect of MDS-UPDRS III, Movement Disorder Society's Revision of the Unified Parkinson's Disease Rating Scale III (MDS-UPDRS III) scores on cerebellar functional connectivity (FC). There were significant main effects when seeding the cerebellar nodes of the frontoparietal (cFPN) and default mode network (cDMN). The color bar represents z-scores, with yellow showing where higher MDS-UPDRS III score (i.e., greater motor dysfunction) was associated with higher FC. **(A)** In Parkinson's disease patients, higher MDS-UPDRS III scores were associated with lower FC between the cFPN and the left superior frontal gyrus (*p* = 0.04, purple). **(B)** Higher MDS-UPDRS III scores were also correlated with lower FC between the cDMN and the right precentral gyrus (*p* = 0.03, purple).

Significant effects of MDS-UPDRS III score were discovered on the FC of cFPN and cDMN. Higher MDS-UPDRS III scores (i.e., higher motor dysfunction) were associated with lower FC between the cFPN and the left superior frontal gyrus and precentral gyrus (*p* = 0.04, purple cluster in Figure 3A). A similar correlation was also present between MDS-UPDRS III scores and FC of the cDMN to the right precentral gyrus and middle frontal gyrus (*p* = 0.03, purple cluster in Figure 3B). No significant effects of MoCA score on cerebellar resting-state node FC were uncovered.

### Interaction Between Montreal Cognitive Assessment Scores and Group

**Figure 4:**
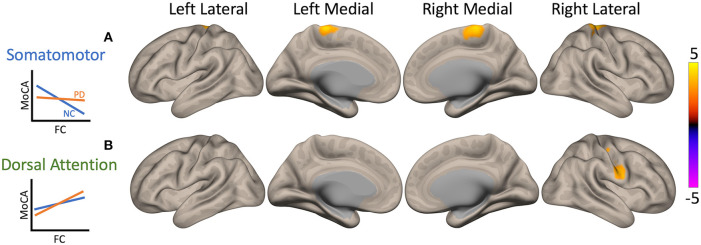
Maps of interaction of Montreal Cognitive Assessment (MoCA) scores and patient condition on cerebellar functional connectivity (FC). Two clusters showed significant interactions when seeding the cerebellar nodes of the somatomotor network (cSMN) and dorsal attention network (cDAN). The color bar represents z-score, with yellow showing where Parkinson's disease (PD) patient correlation values were greater than normal control (NC) participant values. Interaction plots are presented on the left of the brain maps. The y-axis is MoCA score, and the x-axis is FC. PD patients are plotted in orange and NCs in blue. **(A)** In both PD and NC participants, higher MoCA scores (i.e., better cognitive functioning) were associated with lower FC between the cSMN and primarily right precentral gyrus, but PD patients had a weaker correlation compared to controls (*p* = 0.0004, yellow). **(B)** Higher MoCA scores were also associated with higher FC between cDAN and right postcentral gyrus and precentral gyrus; however, PD patients showed greater correlation compared to NC participants (*p* = 0.02, yellow).

In both PD and NC participants, higher MoCA scores (i.e., better cognitive functioning) were associated with lower FC of cSMN to bilateral precentral gyrus, postcentral gyrus, SMA, and right superior parietal lobule (yellow cluster in Figure 4A). However, the correlation value in PD patients was significantly lower than that in NC participants (*p* = 0.0004). Higher MoCA score were also associated with higher FC between the cDAN and the right postcentral gyrus and precentral gyrus in both PD and NC participants (yellow cluster in Figure 4B). This correlation in PD patients was greater compared to NC participants (*p* = 0.02).

## Discussion

This study identified PD-related differences in the FC of cerebellar nodes of RSNs to cortex. As might be expected in a movement disorder, all tests identified clusters that included one or both paracentral lobules. FC levels were correlated with scores on both MDS-UPDRS III and MoCA, providing support for the cerebellum's role in PD.

All clusters identified in our analysis fit with previously discovered regions of dysfunction in PD both with and without cognitive impairment. Paracentral lobules, SMA, frontal gyrus, superior parietal, and occipital lobules have all been identified as areas with abnormal metabolism, connectivity, and/or neurotransmission (2). While occipital dysfunction is not observed consistently, alterations in cholinergic activity that are hypothesized to drive memory deficits are present in the occipital lobe in early PD (31, 32). This is consistent with our finding that there is decreased FC to occipital lobule in our early stage (duration = 7.7 ± 4.2 years) PD cohort. Decreased connectivity between the cerebellum and occipital lobe in PD patients compared to controls has been described previously and associated with visuospatial performance (13, 16). Furthermore, abnormal intracerebellar FC is a common finding in the literature; however, mixed results indicating that this connectivity is both increased and decreased warrant further investigation (8, 13, 15).

In addition, we found that FC between cSMN and somatomotor areas was significantly higher in PD participants compared to that in controls, as described previously (13, 33). FC between these areas was differentially associated with MoCA scores between groups. These differences can be attributed to PD symptoms because both PD and NC groups included participants with cognitive impairment and trends were still present after removal of patients with dementia. Relationships between MoCA score and motor functioning have been reported prior. One study found that motor severity scores significantly predicted worse MoCA scores (34). Additionally, a PD-related pattern of abnormal metabolism, associated with severity of cognitive symptoms, has been identified in patients without MCI or dementia and includes cortical motor areas and parts of the cerebellum (35, 36). On the other hand, we did not detect a significant effect of MDS-UPDRS III scores on FC between these areas. While some studies have reported significant associations between MDS-UPDRS III score and somatomotor cortical FC, others have not discovered this relationship, warranting further investigation of the correlation (13, 15, 17, 37).

The two cerebellar RSN nodes implicated with investigation of the main effect of MDS-UPDRS III score were cFPN and cDMN, both of which showed altered connectivity to somatomotor cortical areas. Similar relationships have been reported before (12, 17). In terms of function, FPN mediates the transition between two contrasting states: a resting state driven by DMN and top-down attention state coordinated by DAN (38). Consistent with these known relationships, lower MDS-UPDRS III scores (i.e., better motor functioning) were associated with higher connectivity between these three networks when seeding the cFPN and cDMN.

The increased connectivity between the cerebellum and somatomotor cortical areas could be explained by the cerebellum's role in error detection and correction for both motor and cognitive functioning (39, 40). The constant error detection and correction occurring in patients with PD would result in strengthened connectivity between the cerebellum and cortex. Some studies have found evidence of a compensatory influence of the cerebellum in PD (17, 41, 42). However, further research is required to solidify the cerebellum's compensatory role (43).

While our findings are congruent with prior research, our ROIs were segregated by their proper RSN rather than traditional cerebellar anatomical structures (12). Since investigation of PD connectivity with a functionally defined cerebellar atlas is novel, future studies should conduct investigations with similar atlases to confirm our results. We chose to use Buckner's 7-network atlas because parcellations were determined by connectivity to large-scale RSNs (12). These networks inherently span multiple functional domains (motor and cognitive) and allowed the investigation of RSNs of interest. Other functionally defined cerebellar atlases should be investigated as well (18, 44, 45).

## Conclusion

This study presents a novel investigation of dysfunction in cerebellar FC to cortex in patients with PD. Analyses conducted with the cerebellum parcellated by cortical RSNs showed FC differences between PD and NC participants. These FC differences were associated with MDS-UPDRS III and MoCA scores for motor and cognitive functioning. Overall, the study provides further evidence for the cerebellum's role in PD.

## Data Availability Statement

The datasets presented in this article are not readily available because data sharing would not comply with institutional review board approval. Requests to access the datasets should be directed to William C. Palmer, wcp23@uw.edu.

## Ethics Statement

The studies involving human participants were reviewed and approved by Institutional Review Board of the University of Washington. The patients/participants provided their written informed consent to participate in this study.

## Author Contributions

SR conceptualized the study. BC and CZ identified the participants and assigned the diagnoses. TM and TG provided the data. WP executed the data analysis with oversight from SR and TG. WP also developed the manuscript. BC, CZ, TG, and SR reviewed and critiqued the manuscript. All authors contributed to the article and approved the submitted version.

## Conflict of Interest

The authors declare that the research was conducted in the absence of any commercial or financial relationships that could be construed as a potential conflict of interest.

## Abbreviations

cSMN: cerebellar components of the somatomotor network
CSF: cerebrospinal fluid
DMN: default mode network
DAN: dorsal attention network
TE: echo time
FWE: family-wise error
FPN: frontoparietal network
FC: functional connectivity
fMRI: functional magnetic resonance imaging
GM: gray matter
TI: inversion time
MCI: mild cognitive impairment
MoCA: Montreal Cognitive Assessment
MDS-UPDRS III: Movement Disorder Society's Revision of the Unified Parkinson's Disease Rating Scale III
NC: normal control
PD: Parkinson's disease
ROI: region of interest
RSN: resting-state network
TR: repetition time
PCA: principal component analysis
SMN: somatomotor network
SMA: supplementary motor area
WM: white matter.
