# Supplementary material for: Resting-State Cerebello-Cortical Dysfunction in Parkinson's Disease
Source: Front Neurol. 2021 Jan 28;11:594213. doi: 10.3389/fneur.2020.594213 (PMC7876057; doi:10.3389/fneur.2020.594213)
Supplement: Supplementary file 2 [file Data_Sheet_1.docx]

**Supplementary Figures**

**
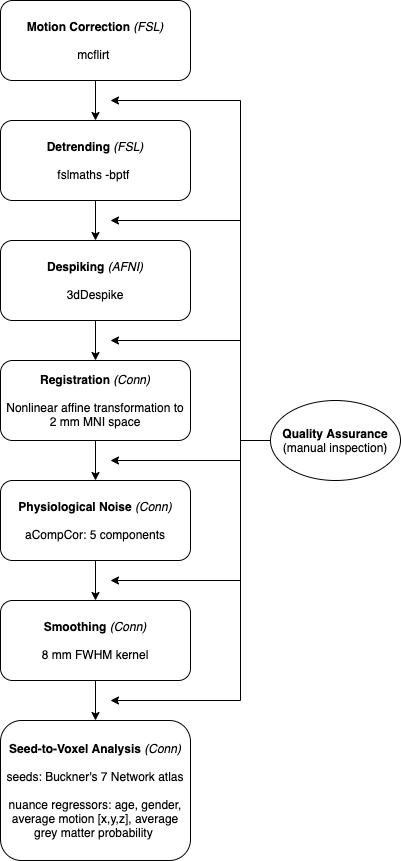
**

**Supplementary Figure 1.** *Flowchart of processing steps for functional images. Diagram shows software and specific commands used to accomplish each step of processing. The quality of each step was manually inspected.*

**
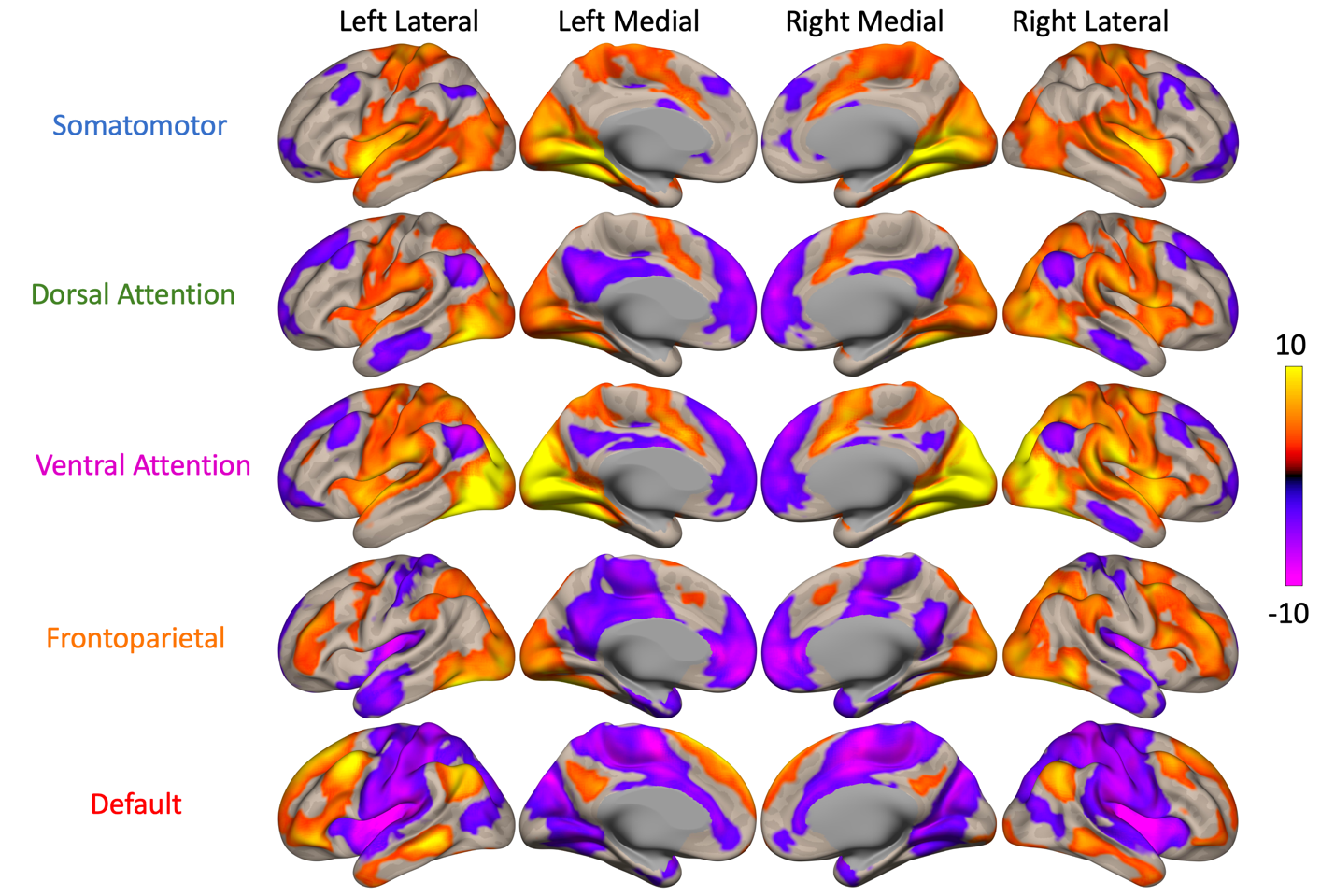
**

**Supplementary Figure 2.** *Average functional connectivity (FC) of each resting-state cerebellar region of interest.* *Maps of average FC when seeding the cerebellar nodes of each resting-state network using a 2-sided voxel-wise thresholding at p=0.005 and cluster-size family-wise error (FWE) correction for multiple comparisons at p=0.05. The color bar represents z-score with yellow displaying positive correlations. Buckner’s parcellations were created by seeding the resting-state networks and investigating their FC to the cerebellum. Seeding each cerebellar node reliably reproduced the resting-state network.*


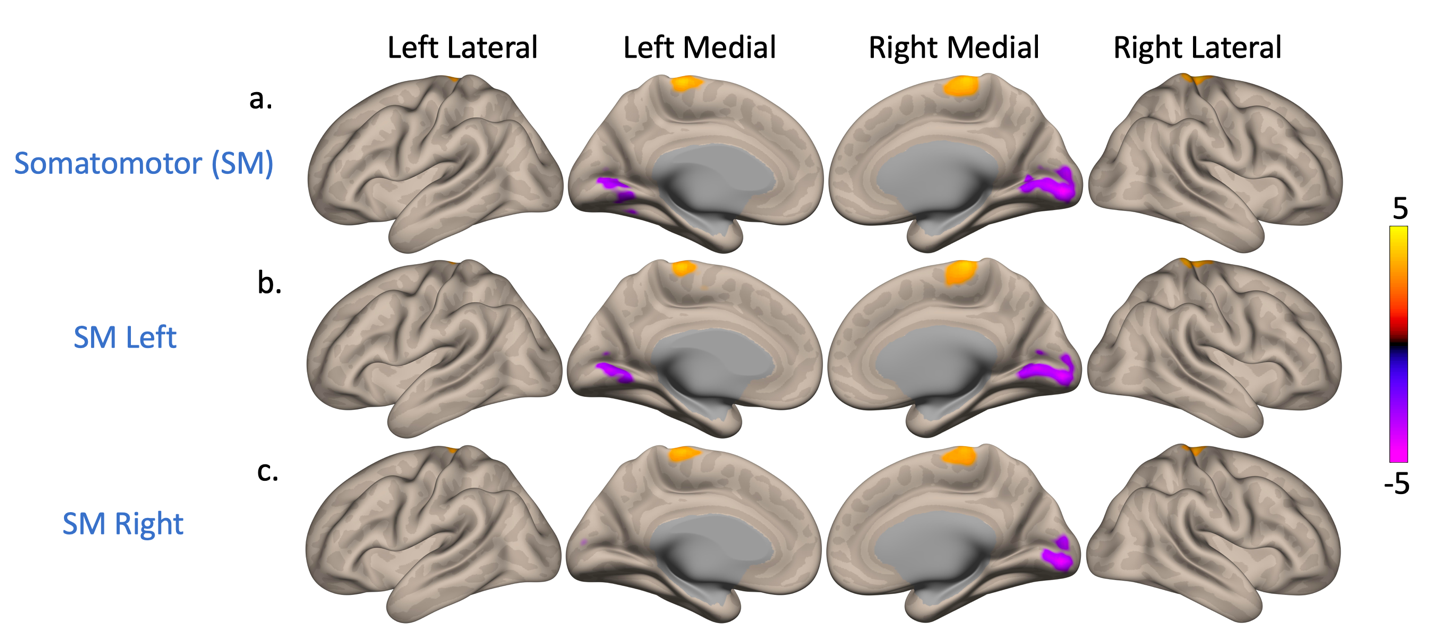


**Supplementary Figure 3.** *Somatomotor cerebellar hemisphere functional connectivity differences between Parkinson’s disease (PD) patients and controls (NC). Maps of connectivity differences when seeding the cerebellar nodes of the somatomotor network (cSMN) and individual hemispheres of the cSMN. The color bar represents z-score with yellow showing where PD functional connectivity (FC) was higher than control FC.* *Clusters from seeding the entire cSMN (****a.****) and left hemisphere (****b.****) produced similar clusters around the precentral gyrus (yellow, p=0.01; p=0.01) and right intracalcarine cortex (purple, p=0.0004; p=0.005).* ***c.*** *However, for the right hemisphere these clusters (yellow, p=0.06; purple, p=0.07) were only evident after reducing the cluster-size FWE thresholding to 0.07.*
